# The Anti-EMMPRIN Monoclonal Antibody hMR18-mAb Induces Tumor Dormancy and Inhibits the EMT Process in Human Carcinoma Cell Lines Co-Cultured with Macrophages

**DOI:** 10.3390/biomedicines13122950

**Published:** 2025-11-30

**Authors:** Elina Simanovich, Felix Oyelami, Phillipp Brockmeyer, Michal A. Rahat

**Affiliations:** 1 Immunotherapy Laboratory, Carmel Medical Center, Haifa 3436212, Israel; 2 Department of Oral and Maxillofacial Surgery, University Medical Center Göttingen, 37075 Göttingen, Germany; 3 Department of Immunology, Rappaport Faculty of Medicine, Technion-Israel Institute of Technology, Haifa 3525422, Israel

**Keywords:** epithelial-to-mesenchymal transition (EMT), EMMPRIN/CD147, TGFβ, migration, angiogenesis, proliferation, dormancy

## Abstract

**Background**: The epithelial-to-mesenchymal transition (EMT) process is necessary for metastasis as it enables tumor cells’ migration and invasion. In the remote organ, tumor cells can develop into metastatic lesions or arrest their proliferation and become dormant, thus suspending metastatic development. EMMPRIN is a membrane glycoprotein, implicated in cell–cell interactions, proliferation, angiogenesis, and EMT. We asked whether neutralizing EMMPRIN with the new anti-EMMPRIN monoclonal antibody hMR18-mAb can inhibit EMT. **Methods**: We co-cultured tumor cell lines (breast carcinoma MCF-7, MDA-MB-231, or oral squamous cell carcinoma SCC-40) together with U937 monocytic-like cells, with or without hMR18-mAb or its negative control rabbit IgG. **Results**: We demonstrate that depending on the initial state of the cells along the epithelial–mesenchymal axis (E/M axis), co-culture enhanced the EMT process, whereas hMR18-mAb reversed this effect. The co-culture increased EMT-inducer cytokines in all cell lines (by 2.5-fold), while hMR18-mAb reduced them (by ~55–70% in the breast cancer cells and by 81% in the SCC-40 cells). The co-culture reduced E-cadherin (by 2-fold in MCF-7 and SCC-40 cells) and increased vimentin expression (by 2–3-fold in MDA-MB-231 and SCC-40), while hMR18-mAb reverted this effect. Co-culture enhanced proliferation, migration, and angiogenic potential of the tumor cells, while hMR18-mAb reduced these by ~20%, 30–44% and ~60–80%, respectively. Co-culture reduced the standard markers of dormancy (NR2F1, p21, p27) and stemness (SOX2, Nanog) (by 30–60% in MCF-7 and SCC-40), while hMR18-mAb elevated gene expression of these markers (by 1.5–3.5-fold) in all cell lines, pushing the cells towards dormancy. **Conclusions**: We conclude that EMMPRIN is a gatekeeper that prevents cells from entering dormancy, and that hMR18-mAb disrupts this effect. As it is the first antibody shown to induce dormancy in tumor cells and stop the development of metastases, this could become a new therapeutic strategy to prevent and treat metastasis.

## 1. Introduction

Breast carcinoma and oral squamous cell carcinoma (OSCC) are epithelial tumors that share aggressive local invasion, early lymphatic spread, epithelial-to-mesenchymal transition (EMT)-driven dissemination, and frequent pathway alterations (TP53; PI3K/AKT; EGFR/HER), as shown by the cancer genome atlas (TCGA) in breast and head–neck cohorts [1,2]. Globally, breast cancer caused ~685,000 deaths in 2020 and presents de novo metastasis in ~6–10%, with ~20–30% of early-stage cases ultimately relapsing distantly [3,4,5]. OSCC shows cervical nodal metastasis in ~20–40% of clinically early cases (N0) and ~5–15% develop distant metastases. Lip and oral cavity cancers caused ~177,757 deaths in 2020 [2,6,7].

The EMT program is essential for the onset of metastasis, as it enhances the mobility and invasiveness of disseminating cells [8]. It is driven by growth factors and inflammatory cytokines, that can be secreted by different stroma cells in the microenvironment, including tumor-associated macrophages (TAMs) [9] such as tumor transforming growth factor β (TGFβ), epidermal growth factor (EGF), and tumor necrosis factor α (TNFα).

To allow epithelial cells undergoing EMT to detach from their neighboring cells in the primary tumor, the EMT transcription factors (EMT-TFs) (e.g., Snail/SNAI1, Slug/SNAI2, Zeb1 and Twist1) suppress the expression of adhesion molecules (e.g., E-cadherin, claudins) and promote the expression of genes such as vimentin and N-cadherin [10,11]. More matrix metalloproteinases (MMPs) such as MMP-9 are secreted [12], to enable remodeling of the extracellular matrix (ECM), migration through it and invasion to remote organs [13]. Depending on their interactions with the new microenvironment, these colonizing disseminating tumor cells (DTCs) can proliferate to form a metastatic lesion, be eradicated by the immune system, or enter a state of dormancy that may last months or years [14,15]. Conversely, to restore their epithelial properties and ability to proliferate, DTCs must overcome signals that trigger quiescence in the organ environment [16], and undergo the opposite mesenchymal-to-epithelial transition (MET) process. Thus, EMT is reversible and transient, and DTCs are plastic and can simultaneously present both epithelial and mesenchymal markers (hybrid or E/M state) at different degrees [17,18]. Reduced rate of proliferation, increased motility, loss of the expression of the epithelial marker E-cadherin and increased expression of the intermediate filament protein vimentin, and of the EMT transcription factors (EMT-TFs) that suppress epithelial adhesion (e.g., Snail/SNAI1, Slug/SNAI2, Zeb1 and Twist1) are used as markers of the EMT process and a mesenchymal phenotype [10,14]. Dormant DTCs can activate a cellular program that reduces proliferation and arrests cell cycle (cellular dormancy) by enhancing the nuclear receptor subfamily 2 group F member 1 (NR2F1), which can in turn, activate the cyclin-dependent kinases p21 and p27 and the stemness markers SRY-Box Transcription Factor 2 (SOX2) and NANOG [19,20]. A balance between the slow rate of proliferation of DTCs due to insufficient blood supply (angiogenic dormancy), and their apoptotic death due to immune cell pressure (immune-mediated dormancy) represent another mechanism of dormancy termed tumor mass dormancy [21]. In fact, the expression of the EMT-TFs, stemness markers and dormancy markers was shown to be linked, as the EMT process often culminates in induced stemness, and dormant cells arrest their cell cycle but need to maintain stem-like properties to allow their survival and ability to self-renew in appropriate conditions [22].

The extracellular matrix metalloproteinase inducer (EMMPRIN), also known as CD147 or basigin, is a multifunctional transmembrane glycoprotein, which is overexpressed in many types of cancer, especially in advanced tumors and in metastatic cells [23,24]. Its ability to interact with myriads of proteins allows it to take part in many processes. For example, its homophilic interactions with another EMMPRIN molecule on an opposite cell mediates stroma-tumor cells interactions and induces signaling that culminates in the induction of MMPs and VEGF [25,26], rendering it a potent pro-angiogenic factor. Its ability to chaperone the monocarboxylate transporters (MCT-1/4), and to bind to glucose transporter 1 (GLUT-1) and integrins implicates it in lactate efflux and metabolic reprograming [27]. Its binding to CD44 and hyaluronan or to cyclophilin A enhances cell invasiveness, stemness, chemoresistance, proliferation and ability to undergo EMT [28,29]. EMMPRIN is also linked with the EMT process, as it enhances β-catenin phosphorylation and suppresses membranal E-cadherin expression [30], and because of the positive feedback loop that exist between it and TGFβ, a potent driver of the EMT process [31,32].

Since EMMPRIN is involved in many processes that are critical to tumor progression, EMT, and metastasis [33], it represents an attractive target. Generally, antibodies are considered good therapeutic agents, due to their high specificity and affinity to their targets, and their ability to kill tumor cells, especially when conjugated to cytotoxic drugs, and many antibodies are now approved to treat cancer diseases [34]. Several monoclonal antibodies that specifically target EMMPRIN were tested in preclinical mouse models of pancreatic cancer [35,36], CML, hepatocellular carcinoma (HCC) [37], esophageal cancer [38] and non-small-cell lung carcinoma (NSCLC) [39]. However, so far, only Metuximab/Licartin^®^, which is a monoclonal murine ^131^-I-labeled F(ab’)_2_ antibody fragment, is currently approved and only in China for clinical use in patients with advanced HCC, in combination with transcatheter arterial chemoembolization (TACE) [40].

Although the involvement of EMMPRIN in the EMT program has been shown, and its importance as a mediator between tumor cells and TAMs that trigger EMT has been recognized [41], its potential as a therapeutic target to induce dormancy has not yet been demonstrated. Here, we test whether hMR18-mAb, a new rabbit anti-human monoclonal antibody directed against the EC-1 domain of EMMPRIN, can induce dormancy in three human breast and oral carcinoma cell lines co-cultured with a monocytic cell line.

In the interest of full disclosure, we note that MAR is an inventor of hMR18-mAb, covered in the patents “Extracellular Matrix Metalloproteinase Inducer EMMPRIN Peptides and Binding Antibodies” and “Monoclonal Antibodies Targeting the Human CD147/EMPRIN Protein”.

## 2. Materials and Methods

### 2.1. Production of hMR18-mAb

**Figure 1:**
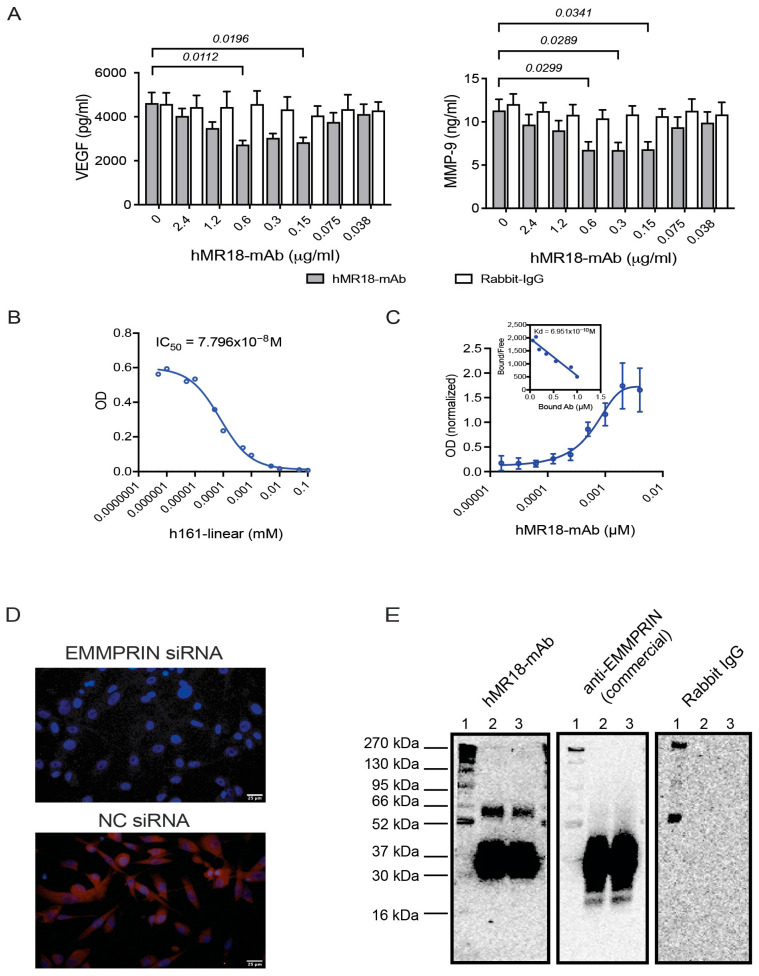
The anti-EMMPRIN hMR18-mAb can specifically recognize EMMPRIN with a good binding affinity. (**A**) A498 cells (4 × 10^5^ cells/800 μL) were co-cultured with U937 cells at a ratio of 2:1, and with TNFα (1 ng/mL) to induce maximal response. The hMR18-mAb or rabbit IgG were added in different concentrations, and cells were incubated for 48 h. At the end of the experiment, supernatants were collected and the concentrations of VEGF and MMP-9 were determined by ELISA (n = 8 for each group). Data are presented as means ± SE and analyzed using the two-way ANOVA followed by Bonferroni’s multiple comparison test. (**B**) Competitive ELISA was carried out as described in the methods. Different concentrations of the linear h161 peptide competed for the binding of the hMR18-mAb antibody to recombinant EMMPRIN (n = 6). (**C**) Cell-based ELISA using fixed A498 cells was performed as detailed in the methods, and the Kd value was calculated from a Scatchard plot (n = 7). (**D**) A498 cells were reverse transfected with EMMPRIN siRNA or with its negative control (NC), as described in the methods. Cells were fixed and then stained with hMR18-mAb. Cells that expressed reduced levels of EMMPRIN were not stained. Bar size is 25 μm. (**E**) Human recombinant EMMPRIN (100 ng/lane) was separated on SDS-PAGE, and transferred to a nitrocellulose membrane. Strips of the membrane were stained with hMR18-mAb, anti-EMMPRIN antibody (Biolegend), or with rabbit IgG. All unmarked p values are insignificant.

We used the same mouse epitope in EMMPRIN that we have previously used [42], which in the human epitope has a difference of 3 amino acids (amino acids position 52–63, GHRWLKGGVVL). To generate the new anti-human EMMPRIN monoclonal antibody, we used the GenScript Rabbit monoclonal development service (GenScript, Jiangning District, Nanjing, Jiangsu, China). The company synthesized the human peptide with an additional cysteine reside, conjugated it to KLH carrier protein and immunized two New Zealand rabbits. After 5 boost injections, serum samples were tested by ELISA for their ability to bind both the peptide and the recombinant EMMPRIN protein. The rabbit with the higher titer was selected, and its splenocytes were fused to a mouse hybridoma cell line to produce hybridomas. These hybridomas were screened, and five positive clones were identified and then sub-cloned by limiting dilutions to ensure the production of stable and positive clones. The clones were then screened in vitro for their ability to inhibit VEGF and MMP-9 in a co-culture system of the human A498 renal cell carcinoma and the human monocytic-like cell line U937. From the five clones tested, only one demonstrated the expected hormetic response in the secretion of MMP-9 and VEGF (Figure 1A), like our previous mouse polyclonal antibody [42] and this clone was selected. The heavy and the light chains were then cloned into the pcDNA3.4 plasmid, expressed in the Expi293F expression system, and the secreted antibody was purified using the Monofinity A Resin Prepacked Column (GenScript). The characterization of the new antibody (hR18-mAb) is described in Section 3.2, in Figure 1 and in Figure S1.

### 2.2. Cell Lines and Co-Cultures

The human breast cancer adenocarcinoma cell line MCF-7 (ATCC HTB-22) was cultured in RPMI-1640 medium with 10% fetal calf serum (FCS), 1% penicillin/streptomycin, 1% pyruvate and 1% L-Glutamine, 1% NEAA, and 10 μg/mL insulin. The human MDA-MB-231 (ATCC HTB-26) adenocarcinoma cell line was cultured in Dulbecco’s Modified Eagle’s Medium (DMEM) medium with 10% FCS, 1% penicillin/streptomycin, 1% pyruvate and 1% L-Glutamine. The human oral squamous cell carcinoma (OSCC) cell line UPCI-SCC-040 (or SCC-40) was obtained from DSMZ (ACC 660) and cultured in MEM with Earle’s salts, 10% FCS, 1% non-essential amino acids, 1% glutamine, and 1% penicillin/streptomycin. The human U937 monocyte-like cells (ATCC CRL-1593) were cultured in RPMI-1640 medium with 10% FCS, 1% penicillin/streptomycin, and 1% amphotericin B. The human renal carcinoma A498 (ATCC HTB-44) was cultured in RPMI-1640 medium with 10% FCS, 1% penicillin/streptomycin, 1% L-Glutamine and 1% non-essential amino acids (NEAA). The human endothelial cell line EaHy926 (ATCC CRL-2922) was cultured in DMEM with 10% FCS, 1% glutamine, 2% HAT (hypoxanthine, aminopterin, thymidine), 1% amphotericin B, and 1% penicillin/streptomycin. All reagents for the tissue cultures were purchased from IMBH- Import and Marketing, Beit Ha’Emek, Israel. All cells were split every 3–4 days at a ratio of 1:4 using trypsin-EDTA, kept in an incubator at 37 °C and 5% CO_2_, and were used at passages 3–15. All cells were routinely checked for the presence of mycoplasma. The A498, MDA-MB-231, MCF-7 and U937 cell lines have been authenticated (Genomic Center of Biomedical Core Facility, Technion).

During the in vitro experiments, cells were seeded in medium that contained only 0.5% FCS, to avoid masking of signals and ensure adherence of the cells to the culture wells. Tumor cells were seeded alone in 24-well plates (4 × 10^5^ cells/800 μL) or in 96-well plates (5 × 10^4^ cells/100 μL) in medium with 0.5% FCS. In co-cultures, the U937 cells that were seeded at half the density as the tumor cells (ratio 1:2) in inserts (0.4 μm pore size, Greiner Bio-One, Kremsmünster, Austria) were added to the 24-well plates with the tumor cells, in the respective tumor cell medium. This prevented cell–cell contact and allowed for the separate extraction of RNA or protein. In some wells, the new hMR18-mAb or the control antibody rabbit IgG (10 ng/mL each) were added to the medium. Wells were incubated for 48 h, and then supernatants were collected, and RNA was extracted. In some experiments, instead of co-culturing, tumor cells were incubated with diluted (as indicated in the figure legends) supernatants collected from previous co-culture experiments, as these supernatants contained all the factors secreted from both cell types, and could represent the co-culture.

### 2.3. Competitive ELISA

We used the competitive ELISA method to characterize the dissociation constant (Kd) of hMR18-mAb to its ligand EMMPRIN. The method is detailed in [43], and relies on using very small concentrations of the reagents (recombinant EMMPRIN and hMR18-mAb) and a monovalent competitor (hapten, the linear h161 peptide) to compete for the binding of the antigen and antibody. The method measures the inhibition of the binding, where in such small concentrations of both the antigen and antibody, the IC_50_ values converge to the Kd values.

We used human recombinant EMMPRIN (100 ng/mL, Biovision, Milpitas, CA, USA) to coat ELISA plates overnight at 4 °C. The plates were washed three times with wash buffer (0.05% Tween 20 in PBS), then blocked with 1% BSA in PBS for 1 h at room temperature and then washed again. Different dilutions (as indicated in Figure 1B) of the h161-linear peptide (sequence: GHRWLKGGVVLC) together with hMR18-mAb (20 ng/mL) were diluted in blocking buffer (0.1% BSA and 0.01% Tween 20 in PBS), added to the wells and incubated for 2 h. After three more washes, the secondary antibody (biotinylated goat anti-rabbit IgG, R&D systems, Minneapolis, MN, USA) was diluted 1:5,000 in blocking buffer and incubated for 2 h. The plate was washed again, and streptavidin-HRP (diluted 1:200 in PBS), was added for 30 min and then washed away three times. Finally, the TMB substrate (Scytek, Logan, UT, USA) was added (100 μL/well) for 20–30 min, the reaction was stopped with 50 μL/well H_2_SO_4_, and OD values were read in the multimode detector microplate DTX 880 (Beckman Coulter, Brea, CA, USA) at 450 nm with reference at 540 nm. Before determining these optimal dilutions of the recombinant EMMPRIN and hMR18-mAb, calibration curves using three different concentrations of each were used (Figure S1) generating a matrix of nine calibration curves and IC_50_ values.

### 2.4. Cell-Based ELISA

A498 cells (7500 cell/well/100 mL) were incubated in full medium overnight, to allow their adherence to the culture plate. Then the cells were washed and fixed in 4% formaldehyde for 10 min, at room temperature. After three washes of the wells with wash buffer (0.05% Tween 20, 1 mM CaCl_2_, 1 mM MgCl_2_ in PBS), plates were blocked with 1% BSA, 0.05% Tween 20 in PBS) for 1 h at room temperature and then washed again three times. The hMR18-mAb (80 mL) was diluted in the blocking buffer and added to the wells for 2 h incubation at room temperature. Plates were washed again three times in wash buffer, and the secondary antibody (biotinylated goat anti-rabbit IgG, R&D systems) was diluted 1:5000 in the blocking buffer and incubated with the fixed cells for 2 h. Plates were washed three times, and streptavidin-HRP (R&D systems) diluted 1:200 in blocking buffer was added for 20 min and then washed three times. TMB (Scytek) was added for 15–20 min, the reaction was stopped with stop solution (50 mL of 1N H_2_SO_4_), and the OD was read in the microplate reader at 450 nm with 540 nm reference. To normalize the OD values to the number of cells, the plates were washed three times and then stained with crystal violet (0.1% crystal violet, 2% ethanol in double distilled water-DDW) for 30 min, cells were washed three times in DDW for 5 min each time, and 1% SDS was added to the cells for 1 h. The OD values were determined in 595 nm. The normalized values were determined by the ratio between the OD values of the ELISA and the OD values of the stained cells.

### 2.5. Sandwich ELISA

The concentrations of TNFα, IGF-1, TGFβ, MMP-9, VEGF, and EMMPRIN in the supernatants of the cultures were determined using the human DuoSet ELISA kits, according to the manufacturer’s instructions (R&D systems). After calibration for each protein, conditioned media were diluted at 1:100 for TNFα, IGF-1, TGFβ, MMP-9, VEGF and EMMPRIN in dilution buffer (1% BSA in PBS). For TGFβ the dilution buffer is 1.4% delipidized bovine serum, 0.05% Tween 20 in PBS. IL-12 diluted 1:10 and IL-10 diluted 1:5 in 1%BSA (in Figure S2). Absorbance was read using the microplate reader (DTX 880 multimode detector, Backman coulter, Brea, CA, USA) at 450 nm with a reference at 540 nm, and final concentrations were calculated according to a standard curve of known protein concentrations. In the case of TGFβ, samples were activated with HCl prior to their addition to the plate, according to the instructions.

### 2.6. Quantitative Real-Time PCR (qPCR)

The Total RNA Purification Kit (Norgen Biotek Corp., Thorold, ON, Canada) was used to extract RNA from the cells according to the manufacturer’s instructions. RNA concentrations were determined by spectroscopy (NanoDrop-One 1000, Thermo Scientific, Waltham, MA, USA). and 3 μg were reverse transcribed using the FIREScript RT cDNA synthesis Mix with oligo (dT) and random primers kit (Solis BioDyne, Tartu, Estonia). Then 80 ng of the cDNA were amplified in triplicates in the StepOne system (Applied Biosystems, Foster City, CA, USA), using the 5X HOT FIREPol EvaGreen qPCR Mix Plus (Solis BioDyne) and 200 nM of the primers (Supplementary Table S1). We used initial activation at 95 °C for 12 min followed by 40 cycles of 95 °C for 15 s, 60 °C for 20 s, and 72 °C for 20 s, to determine the mRNA expression level of the different genes or their endogenous reference gene GAPDH. The relative levels of gene expression were calculated by the comparative ^ΔΔ^CT method according to the formula: RQ = 2^−ΔΔCT^, where ΔC_T_ = C_T_(target gene in reference) − C_T_(GAPDH), and ΔΔC_T_ = ΔC_T_(target gene) − ΔC_T_(calibrator). The non-treated cells served as calibrators in each experiment, and the relative quantity (RQ) between the samples was compared.

### 2.7. Reverse Transfection of A498 Cells to Reduce EMMPRIN Expression

A sterile cover slip was placed in a 12-well plate. The reverse transfection mix was prepared with 50 nM of the EMMPRIN siRNA or its negative control (NC) siRNA (both from Ambion, Thermo Fisher Scientific, Waltham, MA, USA) diluted in 215 mL of Opti-MEM (Gibco, Thermo Fisher Scientific) and placed in each well. Lipofectamine reagent RNAiMAX (Invitrogen, Thermo Fisher Scientific) diluted 1:100 was then added directly to each well containing the diluted siRNA molecules, and the plate was gently mixed to allow equal spreading of the mix. Plates were incubated for 20 min at room temperature. A498 cells (130,000 cells/860 µL full medium/well) were then seeded on top of the mix and incubated for 24 h. After that, the medium was replaced with 1ml of serum starvation medium and incubated for additional 48 h. Cells were then fixed in formaldehyde and expression of EMMPRIN visualized by immunofluorescence.

### 2.8. Immunofluorescence

Tumor cells (5 × 10^4^ cells/400 μL) were seeded on sterile round cover slips that were placed in a 24-well plate and treated according to the experimental procedure. The cells were then fixed with 4% formaldehyde for 10 min, washed 3 times with PBS, and permeabilized (only for the vimentin staining) with 0.25% Triton X-100 in PBS for 10 min. Next, the cells were blocked with blocking solution (2% BSA and 0.1% Triton X-100 in PBS) for 1 h at room temperature, and then incubated overnight at 4 °C with primary antibodies (Rabbit anti-human vimentin diluted 1:750 in blocking solution, Rabbit anti-human E-cadherin diluted 1:150, and Rabbit anti-human EMMPRIN diluted 1:500, all antibodies from Abcam, Cambridge, UK). The cells were washed three times with PBS and then incubated for 1 h at room temperature in the dark with the secondary antibody alexa fluor 555 conjugated donkey anti-rabbit (Abcam) diluted 1:1000 in blocking solution. Cells were washed with PBS for 5 min, incubated in DAPI (100 nM) at room temperature for 5 min and then washed for another 5 min with PBS. Slides were mounted on carrying glass and stored at 4 °C in dark conditions. The same protocol was used for the A498 cells (1.3 × 10^5^ cells) transfected with EMMPRIN siRNA or its negative control, only hMR18-mAb (at 0.48 μg/mL) was used as a primary antibody. Images were taken with the trinocular microscope (Olympus BX-60, Tokyo, Japan) and the MS60 camera and the MShot Image capture System V1 (MSHOT, Guangzhou Micro-shot Technology Co., Guangzhou, China). Analysis of the integrated optical density (IOD) was carried out using Image J software (version 1.54q, National Institutes of Health, Bethesda, MD, USA).

### 2.9. Western Blot Analysis

Recombinant EMMPRIN (100 ng in each lane, Biovision) were loaded on a gradient 4–20% SDS-PAGE, and after separation the proteins were transferred onto a nitrocellulose membrane (Advansta, San Jose, CA, USA). The membrane was then blocked with Block-Chemi reagent (Advansta) for 1 h, and then primary antibodies (hMR18-mAb or mouse anti-human EMMPRIN, Biolegend, San Diego, CA, USA) or Rabbit IgG (Jackson Immuno-Research Labs, West Grove, PA, USA) were diluted in blocking buffer and incubated with the strips of the membrane overnight at 4 °C. The membranes were washed three times in TBST (1xTris-buffered saline with 0.1% Tween 20), and then the HRP-conjugated secondary antibodies (Donkey anti-rabbit IgG or Goat-anti-mouse IgG, Jackson ImmunoResearch Labs, West Grove, PA, USA) were added for 1 h, followed by three additional washes in TBST. Finally, the membranes were incubated with the WesternBright ECL HRP substrate (Advansta), and visualized using the Omega Lum G imaging system (Aplegen, Pleasanton, CA, USA) with the omega Lum G image capture software, version 1.0.

### 2.10. Viability Assay

Tumor cells (10^4^ cells/100 μL) were seeded in 96-well plates and allowed to adhere overnight in full medium. Then the medium was replaced with medium containing only 0.5% FCS diluted 1:1 with conditioned media obtained from the different experimental treatments for 48 h. The CCK-8 solution (10 μL, Abcam, Cambridge, UK) was then added to each well. The plate was allowed to incubate at 37 °C for 2 h. The CCK-8 Cell Viability Assay uses WST-8 tetrazolium salts, which are taken up by all cells and reduced to water-soluble formazan dye in the mitochondria of viable cells only. The absorbance of the reduced product is measured at 450 nm with 620 nm reference and is directly proportional to the number of metabolically active living cells.

### 2.11. Scratch Migration Assay

Tumor cells (5 × 10^4^ cells/70 μL) were seeded at 100% confluence in sterile 96-well plates and allowed to adhere overnight in full medium. Each well was then scratched with the tip of a toothpick, and the detached cells were washed away with PBS. The scratched monolayers were then incubated for 15 h in 80 μL of the conditioned media obtained from the different experimental treatments. The tumor cells were visualized using an inverted microscope. Images were acquired (Moticam 2MP, magnification ×2) immediately after the scratch was washed (T0) and after 15 h (T15). The distance between the two sides of the scratch was measured at multiple points along the scratch using Image Pro plus 4.5 software (Media Cybernetics, Inc., Rockville, MD, USA). The average length at T15 was subtracted from the average length at T0 to determine the length to which the cells migrated.

### 2.12. Wound Healing Assay

The wound assay, assessing the angiogenic potential of cells, was described in detail in [21]. The human endothelial cells EaHy926 (2 × 10^4^ cells/100 μL) were incubated in 96-well plates in full medium overnight to confluency. A scratch was applied with a sterile tip, and detached cells were washed away with PBS. Then, 33 µL of the different experimental conditioned media were diluted 1:2 with the EaHy926 full medium and added to the endothelial cell. Images of the scratch were acquired (Moticam 2MP, magnification ×2) immediately after the wound was applied (T0) and at the end of the experiment (T20). Using the ImagePro plus 4.5 software (Media Cybernetics, Inc., Rockville, MD, USA), the distance between the two sides of the gap at 20 h was subtracted from the distance at 0 h to determine the average distance the endothelial cells proliferated/migrated to.

### 2.13. Statistical Analysis

All experiments were repeated at least five times and results are presented as mean ± SEM (standard error of mean). Statistical significance between three groups or more was determined using one-way ANOVA with Bonferroni post hoc comparison test, and between two groups using the student’s unpaired *t*-test. Significance was achieved at *p*-values less than 0.05 (*p* < 0.05). All statistical analysis was carried out with the GraphPad Prism software for MacOS (version 10.6.1, GraphPad Software, Boston, MA, USA).

## 3. Results

### 3.1. Tumor Cells Induce M2-like Activation of Monocytes and the Co-Culture Enhances EMMPRIN Expression

The ability of tumor cells to reprogram monocytes/macrophages into a M2-like phenotype, which exerts immunosuppression in the tumor microenvironment (TME) and promotes metastasis, has been well documented [44]. To show that this reprograming also takes place in our in vitro system, where the tumor cells are co-cultured with non-activated monocytes (M0) without any additional stimulation, we examined the monocyte phenotypes by assessing M1 and M2 markers. As a positive control for the M2-like phenotype, we incubated the U937 cells with TGFβ, a central and dominant mediator of tumor cells [45]. Relative to their single culture controls, we confirmed that the M1-activation markers CD38, IL-12 and nitrites levels were reduced in the TGFβ-activated U937 cells, as well as in the MCF-7, MDA-MB-231 and SCC-40 cell lines that were co-cultured with the non-activated U937 cells (Figure S2A–C). In contrast, the expression levels of the M2-activtion markers CD206, TGFβ and IL-10 were significantly enhanced by co-culturing the tumor cells with non-activated U937 cells, relative to their single culture controls (Figure S2D–F). Thus, co-culturing non-activated monocytes with tumor cells for 48 h was sufficient to shift the monocytes into a M2-like phenotype.

To learn if the new anti-EMMPRIN monoclonal antibody (hMR18-mAb) can reduce EMMPRIN expression levels, we needed to make sure that EMMPRIN is maximally expressed in the co-cultured tumor cells. Similar to what we have previously shown in other cell lines [25,41,46], EMMPRIN mRNA expression levels in the cell lines used in the current study were unchanged by the co-culture (Figure S3B). However, the EMMPRIN protein levels were increased by about 2-fold, as assessed by the intensity of EMMPRIN immunofluorescent staining of the co-cultured tumor cells relative to the single cultured cells (Figure S3A,C). This suggests that the regulation of EMMPRIN expression is mostly post-translational. Additionally, the secretion of soluble EMMPRIN to the culture media, as assessed by ELISA, was also increased by about 2-fold in the co-cultured cells relative to the single cell cultures (Figure S3D). Of note, the low levels of EMMPRIN secreted by the single cultures of U937 cells demonstrate that the monocytes contribution to the overall soluble EMMPRIN levels is minimal, although some monocytic expression of EMMPRIN is necessary to mediate the homophilic interactions between the two cell types. All this guarantees that the in vitro co-culturing system using the three cell lines is adequate to study the effects of hMR18-mAb.

### 3.2. Validation of the hMR18-mAb Activity

We first characterized the new anti-EMMPRIN monoclonal antibody by determining its IC_50_ value. We took the competitive ELISA approach as described in the methods, using such diluted concentrations of both the recombinant EMMRPIN and hMR18-mAb, where the IC_50_ and the Kd value converge. Using this method, we find that the IC_50_ of hMR18-mAb is 7.796 × 10^−8^ M (Figure 1B), well within the accepted range for monoclonal antibodies. We also measured the Kd using cell-based ELISA, which yields Kd values of the target antigen in its native conformation and quantity. Surprisingly, this Kd value was lower by two orders of magnitude (6.95 × 10^−10^ M) than the IC_50_ value determined by the competitive ELISA (Figure 1C).

To validate that hMR18-mAb can functionally inhibit EMMPRIN activity, we assessed VEGF and MMP-9 secretion in an in vitro system of A498 and U937 cells, using different concentrations of hMR18-mAb or its negative control rabbit IgG. Relative to the secretion of MMP-9 and VEGF in the co-cultured cells, the addition of hMR18-mAb inhibited the secretion, but only between 0.15 and 0.6 μg/mL (Figure 1A). This generated a hormetic dose–response of the antibody, which we have also observed for the mouse polyclonal anti-EMMPRIN antibody (m161-pAb) that we previously reported on [42].

To show the specificity of hMR18-mAb to EMMPRIN, we knocked down EMMPRIN expression in the A498 cells with EMMPRIN siRNA, and immunofluorescently stained them for EMMPRIN with hMR18-mAb. Only the cells transfected with the negative siRNA control were stained (Figure 1D). We also ran a Western blot analysis and observed that hMR18-mAb and the commercially available anti-EMMPRIN antibody recognized human recombinant EMMPRIN, but the rabbit IgG did not (Figure 1E).

### 3.3. The Co-Culture Increases the Secretion of EMT-Inducers, and hMR18-mAb Reduces Their Secretion

**Figure 2:**
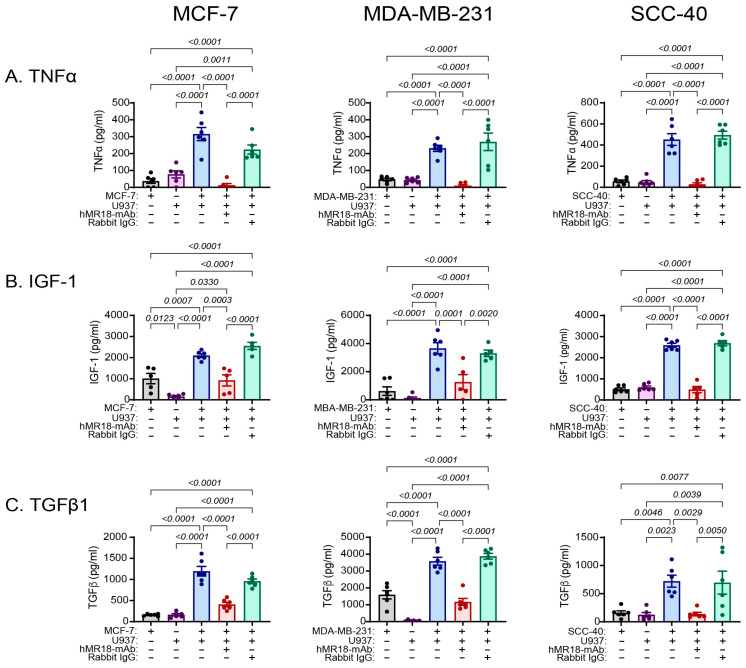
Co-culture enhances and hMR18-mAb inhibits the levels of EMT-inducers. Tumor cells (4 × 10^5^ cells/600 μL) were incubated in a 24-well plate in full medium overnight to allow their adherence. Then the medium was replaced with medium supplemented with only 0.5% FCS, and the cells were incubated alone or with U937 cells (2 × 10^5^ cells) that were seeded in inserts (0.4 μM pore size) for 48 h. In some wells hMR18-mAb or the rabbit IgG (10 ng/mL each) were added to the media. At the end of the incubation, supernatants were collected and the concentrations of (**A**) TNFα, (**B**) IGF-1 and (**C**) TGFβ were determined by ELISA (for all cell lines n = 5–6 in every group).Single tumor cultures, grey bars; Single monocyte cultures, purple bars; Co-cultures, blue bars, addition of hMR18-mAb, red bars; Addition of rabbit IgG, green bars. Data are presented as means ± SE and analyzed using the one-way ANOVA followed by Bonferroni’s post hoc test. All unmarked p values are insignificant.

After the in vitro co-culture conditions were established, we asked which mediators known to induce the EMT program are elevated in the co-cultures, and whether hMR18-mAb can block their elevation. Out of the myriad factors that can trigger the EMT process, we chose to determine the concentrations of three representative EMT-inducers: the potent TGFβ that is strongly associated with the induction of EMT, the pro-inflammatory cytokine TNFα, and the growth factor IGF-1 that activates receptor tyrosine kinases (RTKs) signaling [47]. We show that in all three cell lines, the presence of U937 cells enhanced the secretion of TNFα, IGF-1 and TGFβ relative to each of the single cultures (Figure 2). The addition of hMR18-mAb, but not of the control antibody rabbit IgG, reduced the concentrations of all three cytokines relative to the untreated co-cultures. Thus, all three EMT-inducers are enhanced by the co-cultures and inhibited by hMR18-mAb.

### 3.4. The hMR18-mAb Changes the Expression of EMT Markers

**Figure 3:**
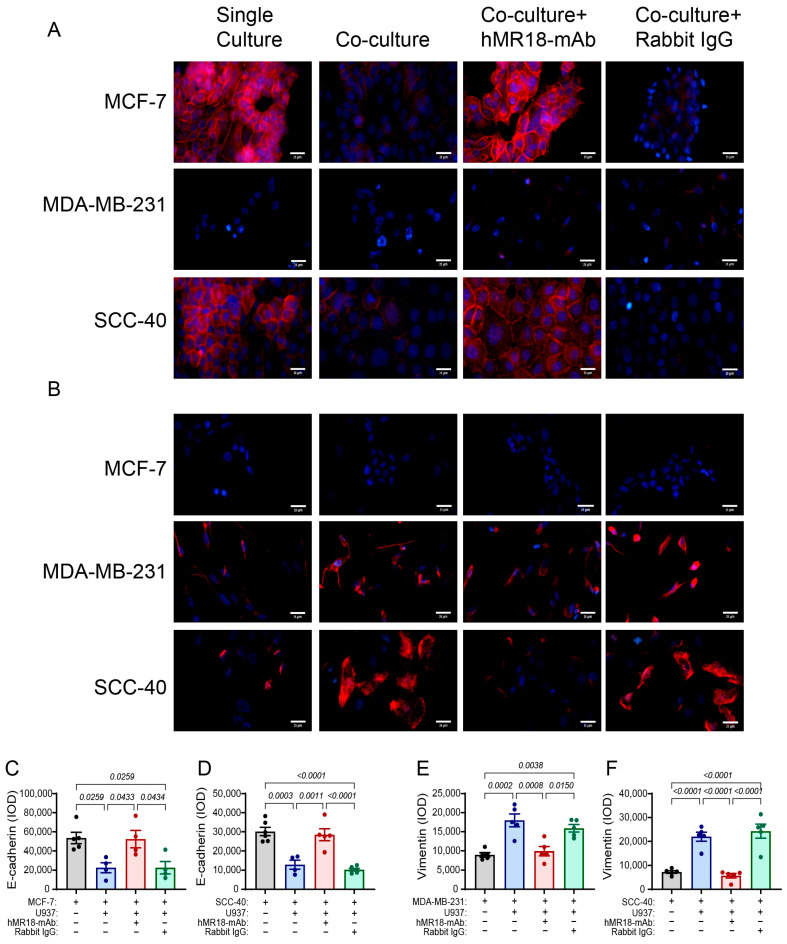
The co-culture affects the expression of the EMT markers E-cadherin and vimentin and hMR18-mAb reverses these effects. The tumor cells lines (5 × 10^4^ cells/400 μL medium supplemented with 0.5% FCS) were incubated on a cover slip alone or co-cultured with U937 monocytes seeded in inserts at a ratio of 2:1 for 48 h. The inserts were removed, and the expression of E-cadherin and vimentin was assessed by immunofluorescent staining. (**A**) Representative images of E-cadherin staining and (**B**) Representative images of vimentin staining. The quantification of the integrated optical density (IOD) of E-cadherin in (**C**) the MCF-7 cell line (n = 4), and (**D**) the SCC-40 cell line (n = 4–5). The quantification of the vimentin IOD in (**E**) the MDA-MB-231 cell line (n = 5), and (**F**) the SCC-40 cell line (n = 4–5). Bar size is 25 μm. Data are presented as means ± SE and analyzed using the one-way ANOVA followed by Bonferroni’s post hoc test. All unmarked *p* values are insignificant.

We next examined the effects of the co-culture and hMR18-mAb on the expression of E-cadherin and vimentin, the two most prominent markers of the epithelial and mesenchymal phenotypes, respectively. The MDA-MB-231 cell line did not express E-cadherin when cultured alone or in co-culture with or without hMR18-mAb (Figure 3A), suggesting that this cell line is already mesenchymal. In contrast, the MCF-7 and SCC-40 cell lines both expressed E-cadherin, whose expression was strongly reduced by 58% during the co-culturing with U937 monocytes (Figure 3A,C,D). Surprisingly, when hMR18-mAb, but not the rabbit IgG, was added to the co-cultured cells, E-cadherin expression was restored in these two epithelial cell lines.

The opposite trend was observed for the expression of the mesenchymal marker vimentin. The MCF-7 did not express any detectable amounts of vimentin under all conditions (Figure 3B), suggesting that this cell line remained with epithelial characteristics despite the co-culturing with U937 monocytes. In both the SCC-40 and the MDA-MB-231 cell lines, vimentin expression was increased in the co-cultures (by 2-fold and 3-fold, respectively), and the addition of hMR18-mAb, but not the rabbit IgG, reduced it (Figure 3B,E,F).

### 3.5. The hMR18-mAb Enhances the Accumulation of mRNAs Coding for the EMT-TFs, Dormancy and Stemness Markers

**Figure 4:**
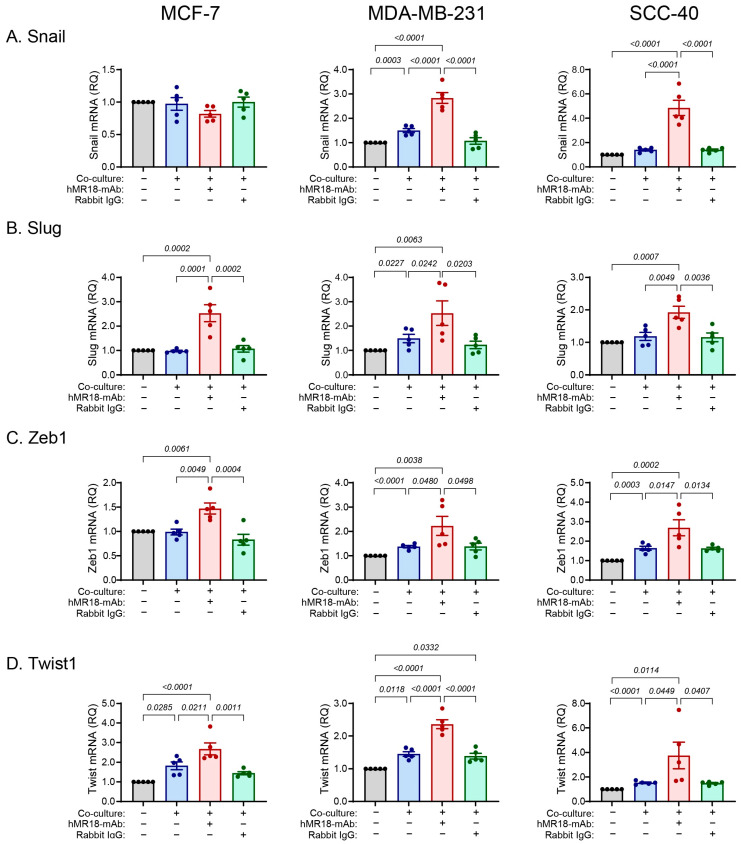
The expression of the EMT-TFs mRNAs is enhanced by the addition of hMR18-mAb. Tumor cells were incubated as described in the legend of Figure 2 with or without the addition of hMR18-mAb or rabbit IgG (10 ng/mL each). After 48 h of incubation, supernatants were aspirated, and total RNA was extracted from the tumor cells, reverse transcribed and amplified by qPCR. In comparison to the non-treated cells that served as calibrators, the relative quantity (RQ) of the mRNA of the EMT-TFs (**A**) *Snail*, (**B**) *Slug*, (**C**) *Zeb1*, and (**D**) *Twist1* was determined in the three cell lines using qPCR (for each cell line n = 5–6 in each group). Data are presented as means ± SE and analyzed using the one-way ANOVA followed by Bonferroni’s post hoc test. All unmarked *p* values are insignificant.

To understand the E/M state of the cells, we looked at the accumulation of mRNAs coding for markers of EMT, stemness and dormancy. First, we looked at the transcription factors known to enhance the EMT process. We show that relative to the single culture, co-culturing with monocytes increased the accumulation of the four EMT-TFs *Snail*, *Slug*, *Zeb1* and *Twist1* in the MDA-MB-231 cell line, whereas in the SCC-40 cells only the accumulation of *Zeb1* and *Twist1* was increased, and in the MCF-7 cell line only the accumulation of *Twist1* was increased (Figure 4). Surprisingly, the addition of hMR18-mAb to the co-culture, but not the rabbit IgG, enhanced the expression of all four EMT-TFs in all three cell lines relative to their co-cultures (by 1.5–3.5-fold), except for *Snail* in the MCF-7 cell line that was not affected.

**Figure 5:**
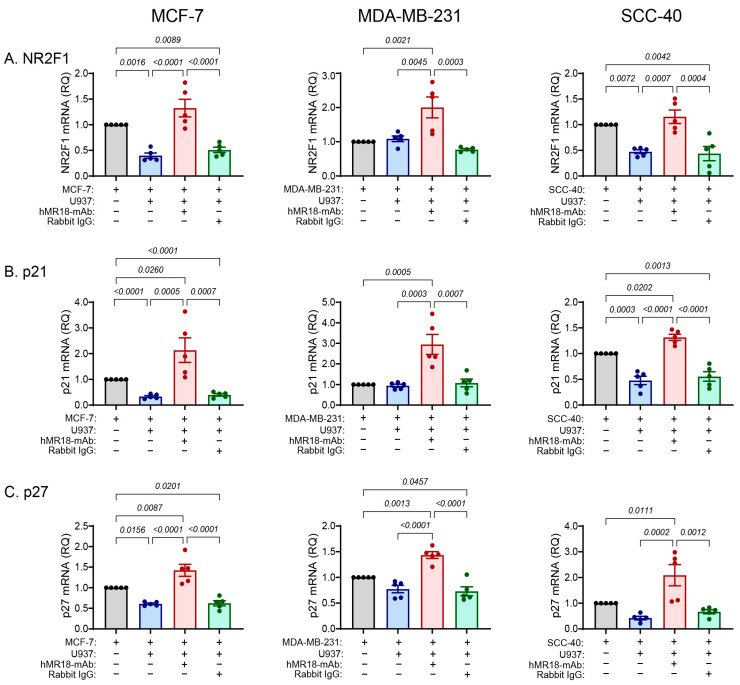
Co-cultures reduced the mRNA coding for the dormancy gene markers, and hMR18-mAb enhanced them. Tumor cells were incubated as described in the legend of Figure 2 and mRNA was extracted, reverse transcribed and amplified as described in Figure 4. In comparison to the non-treated cells that served as calibrators, the relative quantity (RQ) of mRNA of the dormancy markers (**A**) *NR2F1*, (**B**) *p21*, and (**C**) *p27* were determined in the three cell lines (n = 5 in each group). Data are presented as means ± SE and analyzed using the one-way ANOVA followed by Bonferroni’s post hoc test. All unmarked *p* values are insignificant.

In the co-cultures of the MCF-7 and SCC-40 cell lines, the accumulation of mRNA coding for the dormancy markers *NR2F1*, *p21* and *p27* was decreased relative to their single cultures, except for *p27* mRNA in the SCC-40 cell line that showed no significant change. In contrast, the levels of these three mRNAs did not change in the MDA-MB-231 cell line (Figure 5). However, the addition of hMR18-mAb to the co-cultures enhanced the expression of mRNA coding for all dormancy markers in the three cell lines relative to the co-cultures by about 2–5-fold, whereas the rabbit IgG had no effect.

**Figure 6:**
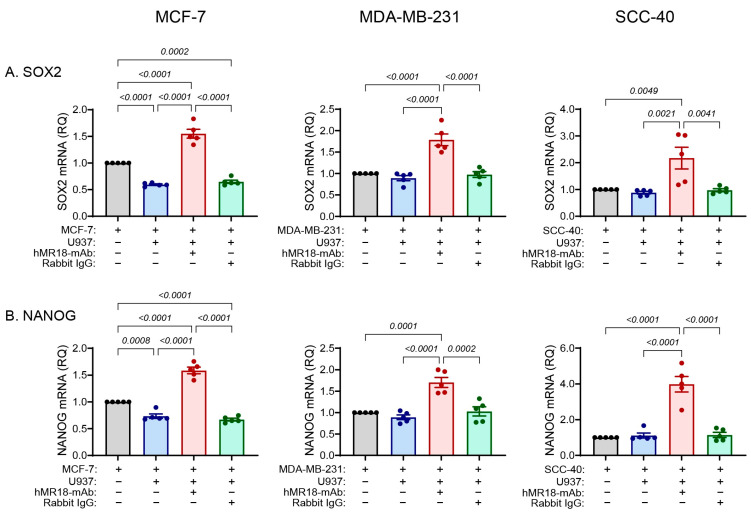
The expression of the stemness gene markers is reduced or unchanged by the co-culture and enhanced by the addition of hMR18-mAb. Tumor cells were incubated as described in the legend of Figure 2, and mRNA was quantified as described in Figure 4. In comparison to the non-treated cells that served as calibrators, the relative quantity (RQ) of the mRNAs of the stemness markers (**A**) *SOX2*, and (**B**) *NANOG* were determined in the three cell lines (n = 5 in each group). Data are presented as means ± SE and analyzed using the one-way ANOVA followed by Bonferroni’s post hoc test. All unmarked *p* values are insignificant.

The mRNA levels for the two stemness markers *SOX2* and *NANOG* were also decreased relative to the single culture of the MCF-7 cell line, whereas in the SCC-40 and MDA-MB-231 cell lines the co-culture did not change their expression (Figure 6). The addition of hMR18-mAb, but not the rabbit IgG, enhanced the expression of *SOX2* and *NANOG* by 2–3-fold in all three cell lines relative to the co-cultures.

### 3.6. The Co-Culture Increases and hMR18-mAb Decreases Proliferation and Migration of Tumor Cells

**Figure 7:**
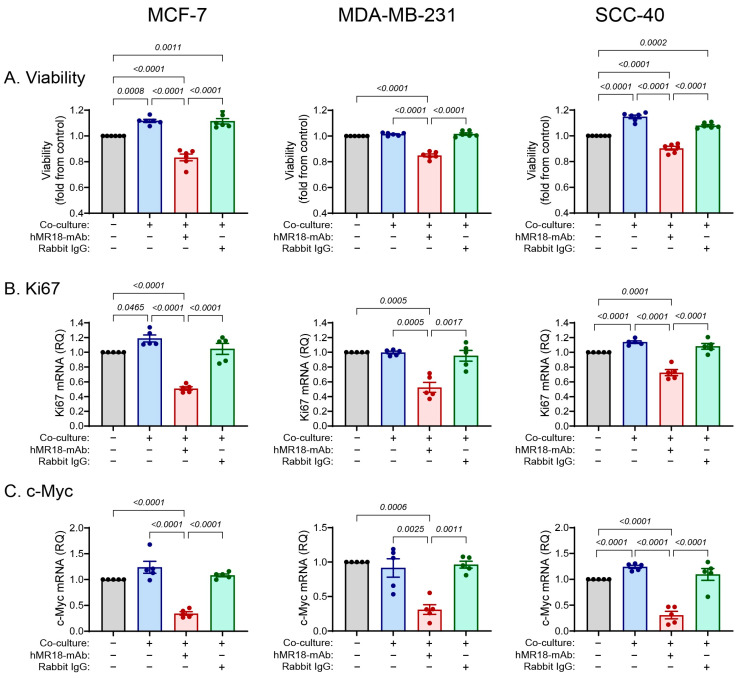
Tumor cell viability is increased by the co-culture, but the addition of hMR18-mAb inhibits it. (**A**) Tumor cells (5 × 10^4^ cells/70 μL) were incubated in 96-well plates in full medium overnight to allow their adherence. Then the medium was replaced with 50 μL the medium supplemented with only 0.5% FCS, and with 50 μL of the conditioned media from the experimental groups, and cells were incubated for 48 h. At the end of the incubation, 10 μL of the CCK-8 reagent were added to each well for 2 h, and the absorbance was determined (n = 6 in each group). Alternatively, total RNA extracted from cells (as described in the legend of Figure 4) was amplified to determine the relative mRNA expression of the proliferation markers (**B**) *Ki67* and (**C**) *c-Myc* (n = 5 in every group). Data are presented as means ± SE and analyzed using the one-way ANOVA followed by Bonferroni’s post hoc test. All unmarked *p* values are insignificant.

Two important properties of cells undergoing EMT are their slow rate of proliferation and increased ability to migrate [14]. By using the CCK-8 viability assay, we show that relative to the single cultures, the co-cultures enhanced the proliferation of the MCF-7 and SCC-40 cell lines by 12% and 15%, respectively, but not of the MDA-MB-231 cell line (Figure 7A). The addition of hMR18-mAb decreased the proliferation of all three cell lines relative to the co-cultures by about 20%, whereas the rabbit IgG had no effect.

A similar trend was observed for the accumulation of the mRNAs coding for the *Ki67* and *c-Myc* proliferation markers [48,49]. The co-cultures enhanced the expression of *Ki67* mRNA in the MCF-7 and SCC-40 cells by 18% and 14%, respectively, and of the *c-Myc* mRNA by about 12%. However, the co-culture had no effect on the expression of these two markers in the MDA-MB-231 cell line. The addition of hMR18-mAb, but not the rabbit IgG, decreased this expression relative to the co-cultures in all three cell lines by more than 40% (Figure 7B,C).

**Figure 8:**
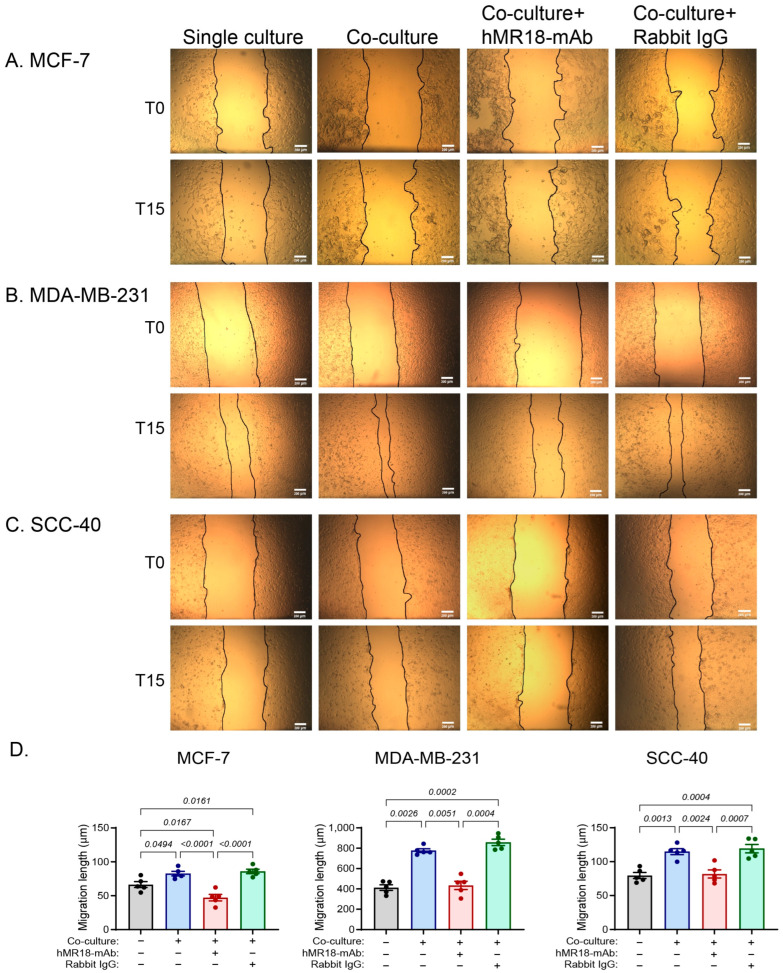
Tumor cell migration is increased by the co-culture, but hMR18-mAb inhibits it. Tumor cells (10^4^ cells/70 μL) were incubated in 96-well plates in full medium overnight to allow their adherence. Then a scratch was applied, the detached cells were washed away, and the adhered cells were incubated with 80 μL of conditioned media obtained from the different experimental groups (as described in Figure 2 and Figure 4). Images were taken at the beginning of the incubation (T0) and after 15 h of incubation (T15). (**A**) Representative images of the MCF-7 cell line, (**B**) Representative images of the MDA-MB-231 cell line, and (**C**) Representative images of the SCC-40 cell line, bar size is 200 μm. (**D**) The length to which each cell line migrated to in order to close the gap was measured as described in the methods (n = 5 for each group). Data are presented as means ± SE and analyzed using the one-way ANOVA followed by Bonferroni’s post hoc test. All unmarked *p* values are insignificant.

Using the scratch assay, the MCF-7, SCC-40 and MDA-MB-231 cell lines demonstrated increased ability to migrate upon co-culturing with the U937 cells, in comparison to the single cultures (by 1.24, 1.44 and 1.88-fold, respectively). However, addition of hMR18-mAb, but not the rabbit IgG, reversed this effect, reducing the migration length back to the levels of the single cultures, or even less in the case of the MCF-7 cells (Figure 8).

### 3.7. The Co-Culture Enhances and hMR18-mAb Reduces the Angiogenic Potential

**Figure 9:**
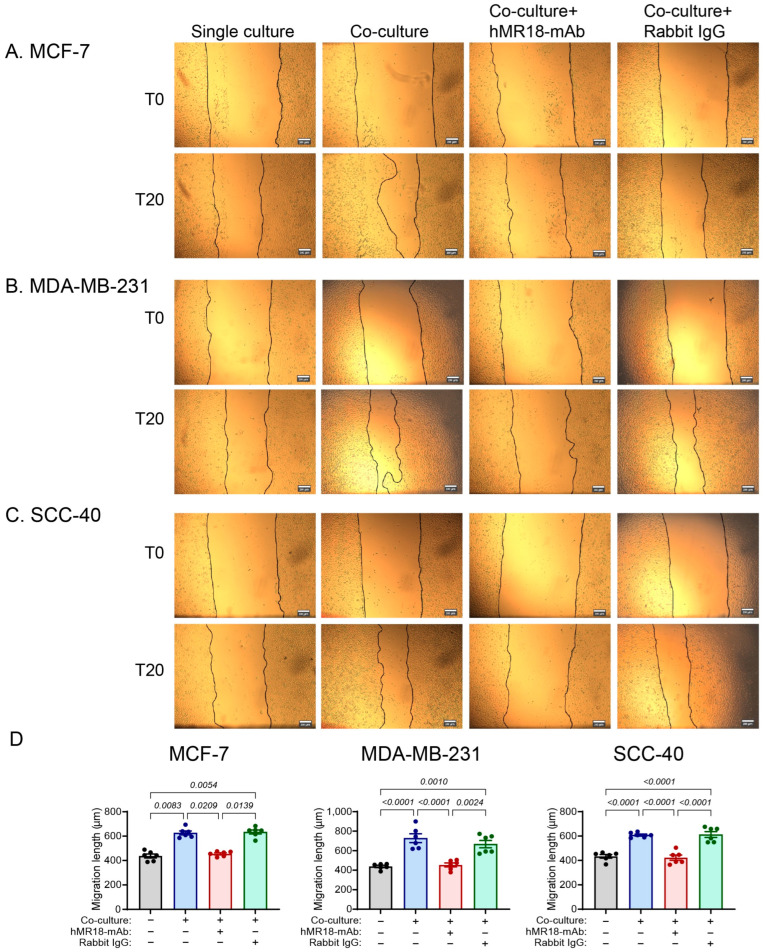
The co-culture increased the angiogenic potential of the EaHy296 endothelial cells, and hMR18-mAb inhibited it. EaHy926 endothelial cells (2 × 10^4^ cells/100 μL) were incubated in 96-well plates in full medium overnight to allow their adherence. Then a scratch was applied, the detached cells were washed away, and the remaining cells were incubated with 67 μL of EaHy926 medium mixed with 33 μL of the conditioned media obtained from the different experimental groups. Images were taken at the beginning of the incubation (T0) and after 20 h of incubation (T20). (**A**) Representative images of the MCF-7 cell line, (**B**) Representative images of the MDA-MB-231 cell line, and (**C**) Representative images of the SCC-40 cell line, bar size is 200 μm. (**D**) Quantitation of the migration length of the EaHy926 cells (n = 5 for each group). Data are presented as means ± SE and analyzed using the one-way ANOVA followed by Bonferroni’s post hoc test. All unmarked *p* values are insignificant.

Angiogenesis is a process that is closely associated with the EMT program. We therefore examined the angiogenic potential induced by the co-culture and the ability of hMR18-mAb to block it. In the wound healing assay, we demonstrate that relative to the single cultures, the conditioned media from the MCF-7, SCC-40 and MDA-MB-231 co-cultures enhanced the ability of endothelial cells to proliferate and migrate, closing the gap (by 1.4, 1.4 and 1.66-fold, respectively, Figure 9). The addition of hMR18-mAb, but not the rabbit IgG, inhibited this response and limited the migration length to that observed in the single cultures.

**Figure 10:**
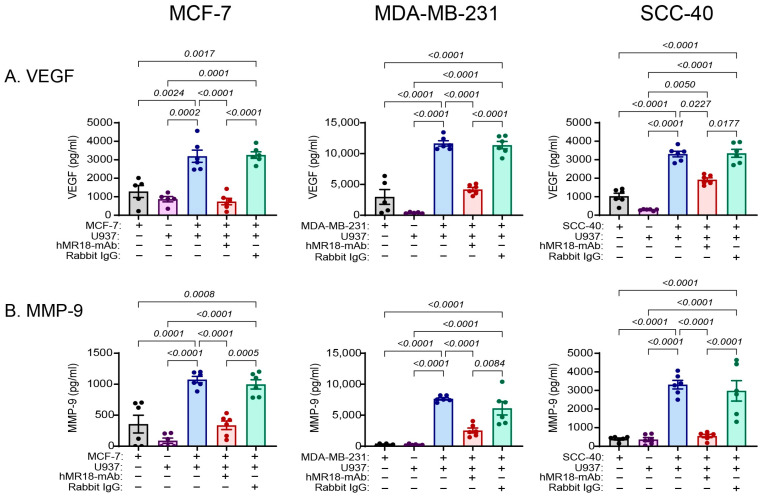
The co-culture enhanced the secretion of pro-angiogenic factors and hMR18-mAb inhibited them. Tumor cells were incubated as described in the legend of Figure 2, with or without the addition of hMR18-mAb or rabbit IgG (10 ng/mL each). After 48 h of incubation the supernatants were collected, and the secreted concentrations of the pro-angiogenic factors (**A**) VEGF and (**B**) MMP-9 were measured using sandwich ELISA (n = 5–6 in each group). Data are presented as means ± SE and analyzed using the one-way ANOVA followed by Bonferroni’s post hoc test. All unmarked *p* values are insignificant.

A similar pattern was revealed for the concentrations of the potent pro-angiogenic factors VEGF and MMP-9 in the conditioned media. The levels of these factors were increased in the co-cultures relative to their levels in each single culture, and the addition of hMR18-mAb, but not the rabbit IgG, decreased these levels in all three cell lines (Figure 10).

## 4. Discussion

In this study, we have demonstrated the ability of the new anti-EMMPRIN monoclonal antibody hMR18-mAb to inhibit the activation of the EMT program and induce dormancy in three tumor cell lines of breast cancer and oral squamous cell carcinoma (OSCC). This demonstrates that the interaction between tumor cells and macrophages, that is mediated by EMMPRIN, is a feasible therapeutic target.

Metastasis is the leading cause of poor prognosis and tumor-related deaths [50]. The EMT program is essential for metastasis, as it allows tumor cells to detach from the epithelial layers, migrate in the circulation, and colonize remote organs. Immune cell infiltration and presence of inflammatory cytokines promote tumor progression and EMT [9]. Thus, monocytes and macrophages, which can make up to 50% of the tumor mass and are mostly activated as M2-like macrophages, can secrete many of the cytokines associated with EMT, including TGFβ, TNFα and IGF1 [51]. Our results confirm that U937 monocytes are activated as M2-macrophages when co-cultured with the tumor cells in vitro, even in the absence of any other stimulation (Figure S2), and that this interaction between the two cell types enhances EMMPRIN expression in the tumor cells (Figure S3). We also show in vitro that this bidirectional interplay between the tumor cells and TAMs is sufficient to promote EMT, while in vivo, other cell types, such as fibroblasts or endothelial cells, may also contribute to the inflammatory milieu and to metastasis. We focused only on the effects of the EMMPRIN-mediated co-culture on the EMT program and the state of dormancy in the tumor cells and did not explore the effects on the U937 cells themselves, although they also express EMMPRIN but to a lesser degree. The effects of hMR18-mAb on the U937 cells and their mode of activation merit a separate, in-depth study.

Generally, we show that the co-culture promoted the EMT program in all the parameters we examined, depending on the relative position of the tested cell line along the E/M axis, while hMR18-mAb pushed the three cell lines into a state of dormancy.

First, we characterized some important properties of hMR18-mAb (Figure 1). The affinity of the antibody to EMMPRIN was evaluated by two methods, and we found that the measured IC_50_ and Kd values differed by two orders of magnitude. The discrepancy could be attributed to differences in EMMPRIN concentrations, conformation, interactions with other proteins during fixation of the cells, or even post-translation modifications between the two methods. However, both methods represent a binding affinity in the nanomolar range, which is considered good for monoclonal antibodies [52]. A hormetic dose–response, observed as a U-shaped graph, has been described before for anti-angiogenic drugs, such as anti-VEGF and integrin inhibitors, without yet being fully explained [53] and is observed in the inhibitory effect of hMR18-mAb. Specificity of hMR18-mAb to EMMPRIN in the cellular context was demonstrated by knocking down EMMPRIN expression with siRNA, resulting in the lack of staining of these cells. Collectively, we validated that hMR18-mAb can specifically recognize human EMMPRIN with good affinity and inhibit its activity.

We monitored three key EMT-inducing cytokines, TGFβ, TNFα and IGF1 (Figure 2), although changes in the levels of other cytokines (e.g., IL-6, IL-8, EGF), could also promote EMT. These three cytokines have been shown to induce the expression of the EMT-TFs and promote the EMT program in different cancer types [54,55,56], especially TGFβ, a major driver of the EMT program that can induce the expression of the EMT-TFs via both its canonical and non-canonical signaling pathways. The co-culture enhanced the secretion of all three cytokines, and hMR18-mAb reduced their levels (Figure 2), suggesting that EMMPRIN takes part in their regulation. The reduction in TGFβ could be explained by the positive feedback loop that exists between EMMPRIN and TGFβ [31,32,57]. However, only few studies suggest the direct involvement of EMMPRIN in the regulation of IGF1 or TNFα. EMMPRIN and IGF1 were suggested to maintain a positive feedback loop [58], and TGFβ was shown to promote IGF1 expression [59]. EMMPRIN could enhance TNFα secretion from monocytes co-cultured with activated platelets [60] and TGFβ was shown to increase TNFα levels in co-cultures of colon carcinoma cells with normal colon epithelial cells, myofibroblasts, and endothelial cells [61]. Thus, we suggest that by neutralizing EMMPRIN, hMR18-mAb leads to reduced TGFβ levels and indirectly to the reduction in the secreted levels of TNFα and IGF1, suggesting that EMMPRIN is involved, either directly or indirectly [60], in the regulation of the complex cytokine network that is generated by the bidirectional interactions of co-cultured tumor cells and monocytes/macrophages.

The reduced expression of E-cadherin and increased expression of vimentin mark the promotion of the EMT program in our co-cultures (Figure 3). The two exceptions were the MDA-MB-231 and the MCF-7 cell lines, which did not express any detectable levels of E-cadherin or vimentin, respectively, and these results were not affected by the co-culture or the addition of hMR18-mAb. This places these two cell lines in the two respective ends of the E/M axis. Conversely, the SCC-40 cells that expresses both E-cadherin and vimentin represents a hybrid phenotype. While the co-culture enhanced the EMT program, resulting in reduced E-cadherin in the MCF-7 and SCC-40 cell lines and increased vimentin in the SCC-40 and MDA-MB-231 cell lines, hMR18-mAb reversed these results. This may suggest an involvement of EMMPRIN in the regulation of E-cadherin expression. A previous study showed that in hepatocytes, overexpression of EMMPRIN induced E-cadherin ubiquitination and degradation and released β-catenin to be translocated into the nucleus [62]. Another study demonstrated that in an endometrial adenocarcinoma cell line, the protein cellular apoptosis susceptibility protein (CAS) stabilizes a complex between E-cadherin and β-catenin, and overexpressed EMMPRIN disrupted this complex, promoting E-cadherin degradation and release of β-catenin, leading to increased EMT [63]. Similarly, EMMPRIN has been implicated as a regulator of vimentin expression, through the activation of the MAPK-ERK signaling pathway [64]. Thus, it may be possible that hMR18-mAb could lead to the stabilization of the E-cadherin membrane complex and to the reduction in MAPK signaling.

The relative position of the cell lines along the E/M axis is further corroborated by the patterns of proliferation and migration. The co-culture increased the proliferation of the epithelial or hybrid cells MCF-7 and SCC-40, whereas the mesenchymal MDA-MB-231, that probably has a very low rate of proliferation, was not affected (Figure 7). Likewise, the co-culture enhanced the migration of the cells at a rate that depends on their respective location along the E/M axis, where the MCF-7 cells, which are more epithelial, exhibited the lowest rate of migration and the MDA-MB-231 cells, which are more mesenchymal, exhibited the highest rate (Figure 8).

While EMT is usually associated with increased migration and decreased proliferation, our results that show both increased migration and increased proliferation are, in fact, in agreement with numerous other studies [65,66,67]. The discrepancy could arise from the specific hybrid EMT phenotype of the cells, from the acquisition of stem cell properties that maintain slow proliferation rate of the cells, or from the presence or absence of specific microenvironmental signals (e.g., hypoxia, cytokines secreted from other cell types) that differently affect proliferation. Nonetheless, hMR18-mAb reverted the EMT-inducing effects of the co-culture on both proliferation and migration in all three cell lines, demonstrating the involvement of EMMPRIN in these processes.

Another corroboration for the status of the three cell lines along the E/M axis is reflected in the ability of the co-culture to activate the transcription of the EMT-TFs that execute the EMT program. We observed a gradual involvement of the EMT-TFs, where the co-culture increased only *Twist1* mRNA in the MCF-7 cells, *Twist1* and *Zeb1* mRNAs in the SCC-40 cells, and all four EMT-TFs in the MDA-MB-231 cells (Figure 4). A similar but opposite gradual involvement is observed for the expression of the three dormancy marker genes in the co-cultures. The co-culture reduced the expression of the *NR2F1*, *p21* and *p27* mRNAs in the MCF-7, but only of the *NR2F1* and *p21* mRNAs in the SCC-40 cell lines, whereas none of the dormancy markers was affected in the MDA-MB-231 cell line (Figure 5). Likewise, the stemness mRNAs coding for *SOX2* and *NANOG* were reduced only in the MCF-7 cell line (Figure 6). However, regardless of the relative positions of the cells along the E/M axis, the addition of hMR18-mAb to the co-cultures increased the expression of all these genes in all three cell lines relative to the untreated co-cultures.

This may implicate EMMPRIN in the regulation of the EMT-TFs and could suggest a link between these sets of genes and the processes of EMT [22]. For example, elevated levels of *Zeb1* and *Snail* can attenuate cell proliferation by inhibiting the expression of PCNA, cyclin D1 and D2, and the phosphorylation of retinoblastoma (Rb) [68]. *Snail* and *Slug* were shown to downregulate the expression of cyclin D2 and induce the expression of p21 and p27 to inhibit cell proliferation [11,69]. As dormancy is defined by the ability of cells to arrest their cell cycle and their ability to self-renew (stemness), which can be reversed upon receiving the correct signals from the niche, these examples link the EMT program to quiescence. On the other hand, CDK4/6 can stabilize *Zeb1* by activating its de-ubiquitinase USP51, thus promoting cell migration and invasion [70], and p27 can enhance *Twist1* expression [71], linking dormancy to the EMT program, and demonstrating the Go-or-Grow principle, whereby a cell located on the extreme ends of the E/M axis can either proliferate or migrate [72]. Based on these links and interactions, we propose that the balance between the expression of these three sets of genes determines the state of the cell. If only EMT-TFs are activated but the stemness and dormancy genes are suppressed, as seen in the co-cultures, the EMT program is activated, and the cells gain a mesenchymal phenotype. But when hMR18-mAb is applied and all three sets of genes are induced, then the cells become dormant and proliferation is downregulated.

While EMT and angiogenesis are two separate processes, they are closely intertwined. EMT inducers, such as TGFβ, IL-6, IL-8, and TNFα, are also pro-angiogenic factors [73,74], and EMT-TFs can induce the expression of VEGF and MMP-9 [75,76,77]. Conversely, VEGF and other pro-angiogenic factors, such as fibroblast growth factor-2 or angiopoietin-2, can promote the EMT program by inducing the expression of the EMT-TFs, and promoting cell migration through the rearrangement of the cytoskeleton and induction of MMPs, as reviewed in [77]. In addition, hypoxia, which results from the expansion of tumors and is known as a strong inducer of angiogenesis, also induces the expression of the EMT-TFs [77]. Moreover, angiogenesis provides tumor cells with new routes of exit to the circulation [77,78]. However, when angiogenesis is reduced or not activated, and tumor cells do not receive a sufficient supply of oxygen and nutrients to support their proliferation, angiogenic dormancy may follow. We show that conditioned media of co-cultured cells from all three cell lines increased the angiogenic potential of the cells, as was manifested by the increased migration of the EaHy926 endothelial cell line (Figure 9), and elevated concentrations of the pro-angiogenic factors VEGF and MMP-9 (Figure 10). Addition of hMR18-mAb to the co-culture inhibited this effect, supporting the antibody’s ability to initiate angiogenic dormancy.

## 5. Conclusions

In conclusion, by applying hMR18-mAb, we demonstrated the involvement of EMMPRIN, either directly or indirectly, in several functions that are critical for the EMT program and for the progression of metastasis. Therefore, EMMPRIN becomes a very attractive candidate for targeting. Targeting EMMPRIN with hMR18-mAb, thus pushing DTCs in remote organs to a state of dormancy, could be used in the clinics to treat metastasis and perhaps even to prevent it. This would require further testing of hMR18-mAb using in vivo mouse models, with or without combinations with chemotherapy or immunotherapy before applying this new approach to human patients.

## Data Availability

The raw data supporting the conclusions of this article will be made available by the authors on request.

## Supplementary Materials

The following supporting information can be downloaded at: https://www.mdpi.com/article/10.3390/biomedicines13122950/s1. Table S1: List of primers used for qPCR amplification. Figure S1: Calibration of the range of reagents needed for the determination of the IC_50_ values for hMR18-mAb. Figure S2: Tumor cells can shift monocyte activation towards M2-like phenotype. Figure S3: Co-culturing with monocytes enhances EMMPRIN protein expression and secretion.

## Abbreviations

The following abbreviations are used in this manuscript:

BMP	Bone morphogenetic protein
DTCs	Disseminating tumor cells
ECM	Extracellular matrix
EMMPRIN	Extracellular Matrix metalloproteinase Inducer
EMT	Epithelial-to-mesenchymal transition
EMT-TFs	Epithelial-to-mesenchymal transition transcription factors
EGF	Epidermal growth factor
FGF	Fibroblast growth factor
IL	interleukin
GLUT-1	Glucose Transporter Type 1
HCC	Hepatocellular carcinoma
OSCC	Oral Squamous cell carcinoma
PDGF	Platelet-derived growth factor
MCT1/4	Monocarboxylate Transporter 1/4
MET	Mesenchymal-to-epithelial transition
MMP	Matrix Metalloproteinases
NSCLC	Non-small-cell lung carcinoma
NR2F1	Nuclear Receptor Subfamily 2, Group F, Member 1
SCC-40	UPCI-SCC-040
VEGF	Vascular endothelial growth factor
TAMs	Tumor-associated macrophages
TGFb	Transforming growth factor b
TNFa	Tumor necrosis factor a
