# Supplementary material for: The Anti-EMMPRIN Monoclonal Antibody hMR18-mAb Induces Tumor Dormancy and Inhibits the EMT Process in Human Carcinoma Cell Lines Co-Cultured with Macrophages
Source: Biomedicines. 2025 Nov 30;13(12):2950. doi: 10.3390/biomedicines13122950 (PMC12730406; doi:10.3390/biomedicines13122950)
Supplement: Supplementary file 1 [file biomedicines-13-02950-s001.zip › biomedicines-3959068-supplementary.pdf]

Table S1. List of primers used for qPCR amplification.

Gene amplified	Forward primer	Reverse primer
Basigin (EMMPRIN)	5'- TGGCCTTCACGCTCTTGAG-3'	5'- CAACGCCACTGCTGTTCAAA-3'
Snail	5'-TGTCTGCACGACCTGTGGAAAG-3'	5'-CTTCACATCCGAGTGGGTTTGG-3'
Slug	5'-TCTGTGGCAAGGCTTTCTCCAG-3'	5'-TGCAGATGTGCCCTCAGGTTTG-3'
Twist1	5'-GATTCAGACCCTCAAACTGGCG-3'	5'-AGACGGAGAAGGCGTAGCTGAG-3'
Zeb1	5'-ATTCAGCTACTGTGAGCCCTGC-3'	5'-CATTCTGGTCCTCCACAGTGGA-3'
SOX2	5'-AACGGCAGCTACAGCATGATGC-3'	5'-CGAGCTGGTCATGGAGTTGTAC-3'
Nanog	5'-GAACGCCTCATCAATGCCTGCA-3'	5'-GAATCAGGGCTGCCTTGAAGAG-3'
NR2F1	5'-CCAACAGGAACTGTCCCATCGA-3'	5'-CCGTTTGTGAGTGCATACTGGC-3'
p21	5'-TCGCTGTCTTGCACTCTGGTGT-3'	5'-CCAATCTGCGCTTGGAGTGATAG-3'
p27	5-AGCAGTGTCCAGGGATGAGGAA-3'	5'-TTCTTGGGCGTCTGCTCCACAG-3'
c-Myc	5'-TCGCTGCTGTCCTCCGAGTCC-3'	5'-GGTTTGCCTCTTCTCCACAGAC-3'
Ki-67	5'-GAGGAGAAACGCCAACCAAGAG-3'	5'-TTTGTCCTCGGTGGCGTTATCC-3'
GAPDH	5'- CATCACTGCCACCCAGAAGACTG-3'	5'- ATGCCAGTGAGCTTCCCGTTCAG-3'

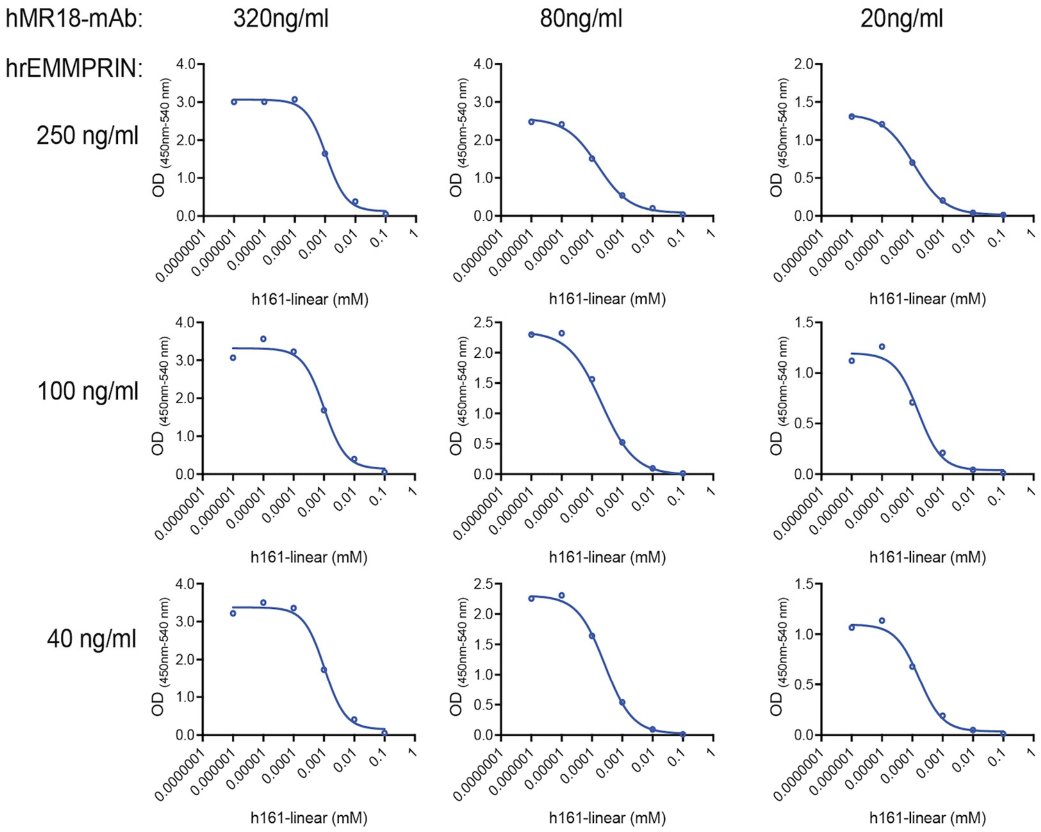

IC50 values	hMR18-mAb (ng/ml)		
**hrEMMPRIN (ng/ml)**	**320**	**80**	**20**
**250**	0.001100	0.000156	0.000114
**100**	0.001019	0.000207	0.000157
**40**	0.001046	0.000266	0.000166

Figure S1. Calibration of the range of reagents needed for the determination of the IC_{50} values for hMR18-mAb. Competitive ELISA was carried out as described in the methods, with three different concentrations of human recombinant EMMPRIN and hMR18-mAb as indicated. This generated a matrix of nine calibration curves with different IC_{50} values for each of the concentrations, summarized in the table. Based on these calibration results, we chose to proceed with a concentration of 100 ng/ml of recombinant EMMPRIN and 20 ng/ml of hMR18-mAb.

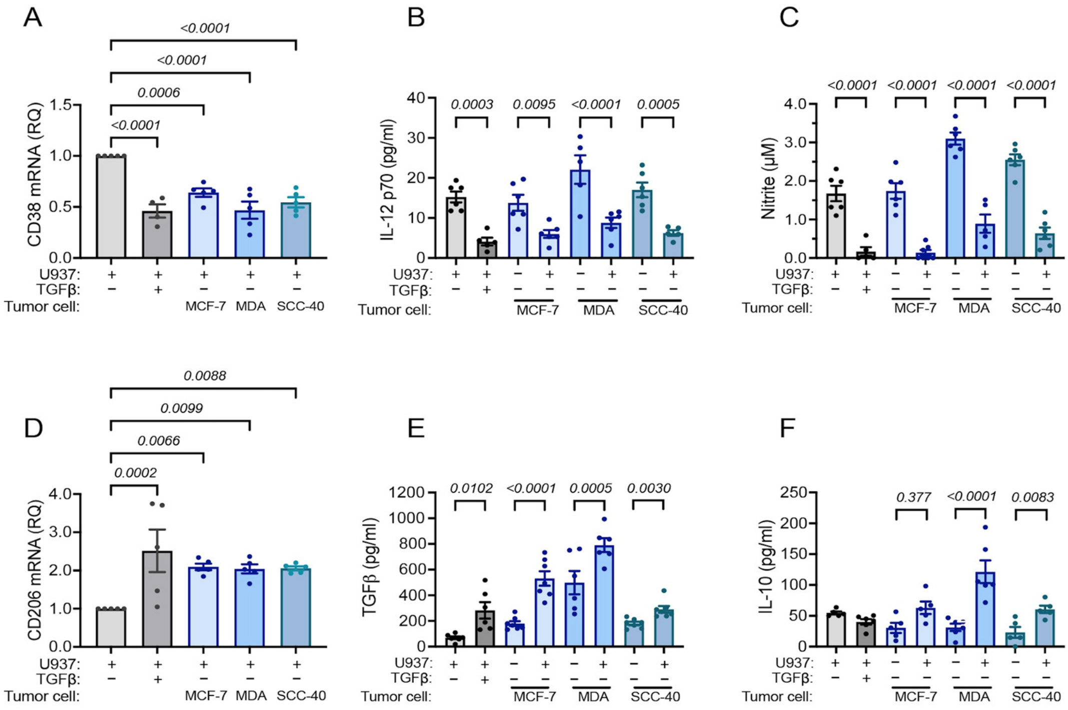

Figure S2. Tumor cells can shift monocyte activation towards M2-like phenotype. Tumor cells (2x10^5 cells/600 µL) were incubated in full medium overnight to allow their adherence. U937 monocytes (2x10^5 cells) were seeded in the upper chamber of the inserts (0.4 µm pore size) and incubated alone (representing M0 activation) or in co-culture with the different tumor cells. As positive control for the M2-activation, TGFβ (5 ng/ml) was added to the U937 cells seeded as single cultures. Cells were then incubated for 48 h in the tumor cell medium with only 0.5% FCS. At the end of the incubation, RNA was extracted from the U937 cells, and the concentrations of cytokines were determined in the conditioned media. The expression levels of the M1-activation markers **(A)** CD38 mRNA (n=4 in U937+TGFβ, n=5 in all other groups), **(B)** IL-12 (n=5 in the U937+TGFβ, MDA-MB-231, and SCC-40+U937 groups, and n=6 in all other groups), and **(C)** nitrites (n=5 in the U937+TGFβ, and MDA-MB-231+U937 groups, and n=6 in all other groups) were reduced by the co-culture, whereas the expression levels of the M2-activation markers **(D)** CD206 mRNA (n=5), **(E)** TGFβ (n=6 in the U937+TGFβ, MDA-MB-231 and MDA-MB-231+U937 groups, and n=7 in all other groups), and **(F)** IL-10 (n=5 in the U937, MCF7, MCF+U937, SCC-40 and SCC-40+U937 and n=6 in all other group) were enhanced. Data are presented as means ± SE and analyzed using ANNOVA followed by the Bonferroni's multiple comparisons test. All unmarked p values are insignificant. .

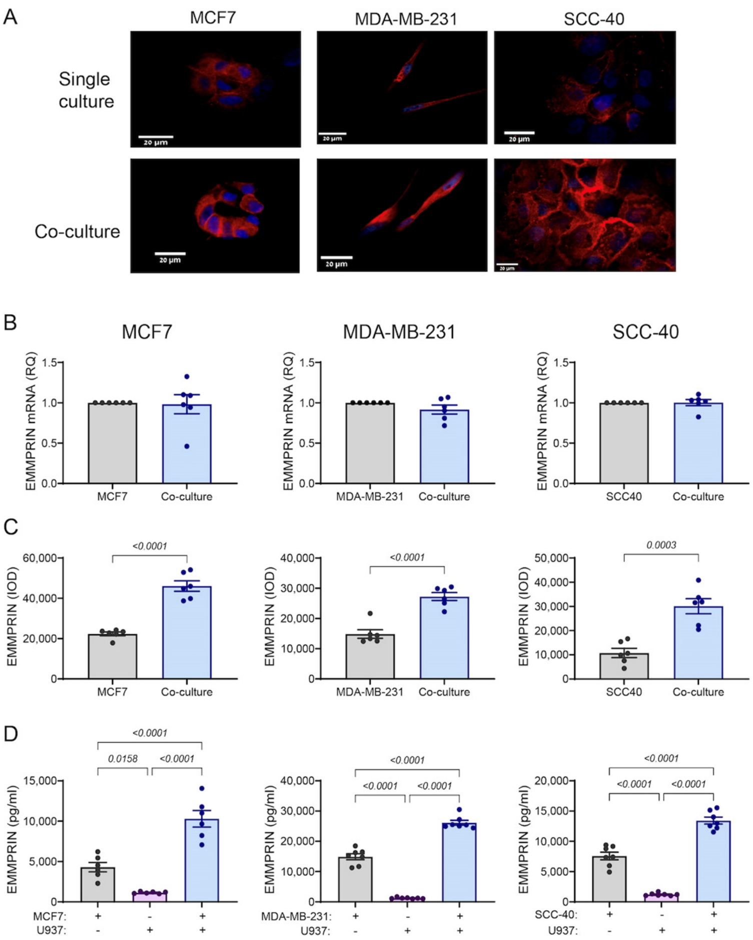

Figure S3. Co-culturing with monocytes enhances EMMPRIN protein expression and secretion. The tumor cells lines (5x10^4 cells/400 μL with 0.5% FCS) were incubated on a cover slip alone or co-cultured with U937 monocytes at a ratio of 2:1 for 48 h and immunofluorescently stained for EMMPRIN protein expression. **(A)** Representative images demonstrating enhanced EMMPRIN protein expression in the co-cultured tumor cells (bar size is 20 μm). **(B)** The tumor cells (2x10^5 cells/600 μL medium with 0.5% FCS) were incubated alone or in co-culture with U937 monocytes at a ratio of 2:1 for 48h. The EMMPRIN mRNA levels in the tumor cells were determined by qPCR (n=6). No change in EMMPRIN mRNA levels was observed. **(C)** The integrated optical density (IOD) of EMMPRIN expression in tumor cells that were cultured alone or with monocytes as depict in (A) (n=6). **(D)** The tumor cells and U937 cells were incubated as described in (B), and the concentrations of the secreted EMMPRIN were determined in the conditioned media by ELISA (n=6 for the MCF7 cells, n=7 for the MDA-MB-231 and SCC-40 cells). Data are presented as means ± SE and analyzed using the two-tailed Student's *t* test analysis when comparing two groups, or with the one-way ANOVA followed by Bonferroni's post-hoc test when comparing three groups. All unmarked p values are insignificant. .
